# Structure, function, and evolution of metallo-β-lactamases from the B3 subgroup—emerging targets to combat antibiotic resistance

**DOI:** 10.3389/fchem.2023.1196073

**Published:** 2023-06-20

**Authors:** Stefan Krco, Samuel J. Davis, Pallav Joshi, Liam A. Wilson, Marcelo Monteiro Pedroso, Andrew Douw, Christopher J. Schofield, Philip Hugenholtz, Gerhard Schenk, Marc T. Morris

**Affiliations:** ^1^ School of Chemistry and Molecular Biosciences, The University of Queensland, Brisbane, QLD, Australia; ^2^ Australian Centre for Ecogenomics, The University of Queensland, Brisbane, QLD, Australia; ^3^ Chemistry Research Laboratory, Department of Chemistry, The Ineos Oxford Institute for Antimicrobial Research, Oxford University, Oxford, United Kingdom; ^4^ Sustainable Minerals Institute, The University of Queensland, Brisbane, QLD, Australia; ^5^ Australian Institute of Bioengineering and Nanotechnology, The University of Queensland, Brisbane, QLD, Australia

**Keywords:** antibiotic resistance, β-Lactamase, Metallo-β-Lactamase superfamily, classification, structure, function, evolution, drug development

## Abstract

β-Lactams are the most widely employed antibiotics in clinical settings due to their broad efficacy and low toxicity. However, since their first use in the 1940s, resistance to β-lactams has proliferated to the point where multi-drug resistant organisms are now one of the greatest threats to global human health. Many bacteria use β-lactamases to inactivate this class of antibiotics via hydrolysis. Although nucleophilic serine-β-lactamases have long been clinically important, most broad-spectrum β-lactamases employ one or two metal ions (likely Zn^2+^) in catalysis. To date, potent and clinically useful inhibitors of these metallo-β-lactamases (MBLs) have not been available, exacerbating their negative impact on healthcare. MBLs are categorised into three subgroups: B1, B2, and B3 MBLs, depending on their sequence similarities, active site structures, interactions with metal ions, and substrate preferences. The majority of MBLs associated with the spread of antibiotic resistance belong to the B1 subgroup. Most characterized B3 MBLs have been discovered in environmental bacteria, but they are increasingly identified in clinical samples. B3-type MBLs display greater diversity in their active sites than other MBLs. Furthermore, at least one of the known B3-type MBLs is inhibited by the serine-β-lactamase inhibitor clavulanic acid, an observation that may promote the design of derivatives active against a broader range of MBLs. In this Mini Review, recent advances in structure-function relationships of B3-type MBLs will be discussed, with a view to inspiring inhibitor development to combat the growing spread of β-lactam resistance.

## 1 Introduction

The spread of antibiotic resistance is arguably one of the greatest current threats to global health, with multidrug-resistant pathogenic strains increasingly encountered in clinical settings (World Health Organization, 2014). Concerningly, resistance is also being discovered in environmental bacteria that seemingly have not yet been subjected to the same levels of selective pressure as medically relevant pathogenic microbes (though antibiotics are very widely used in farming) (Miraula et al., 2016; Selleck et al., 2020; Wilson et al., 2021).

**FIGURE 1:**
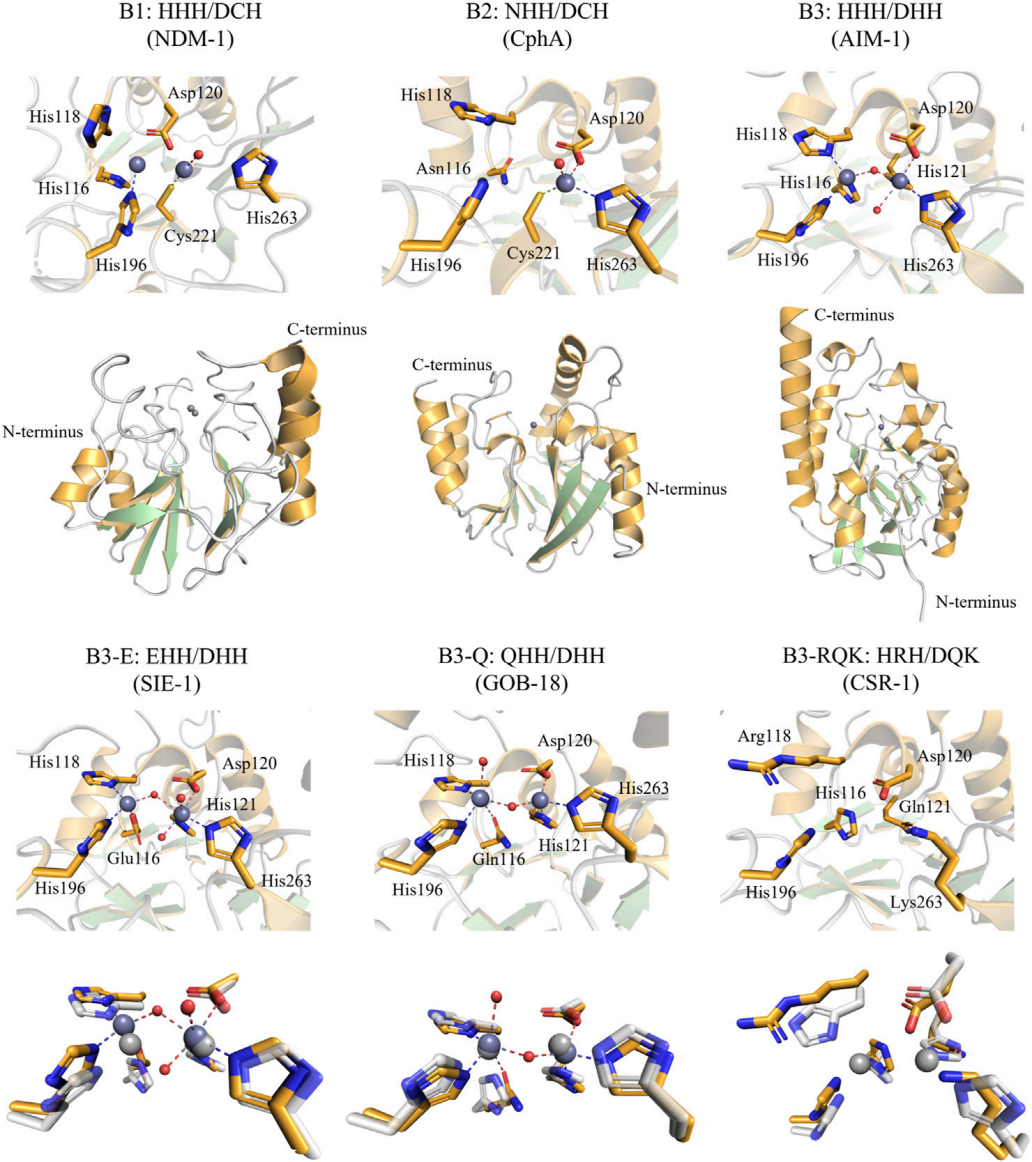
Active site and overall structure views of Class B MBLs (“true MBLs”) from the B1, B2, and B3 subfamilies. Residues are numbered according to the BBL numbering scheme (Garau et al., 2004). Note that the B1 and B3 MBLs employ two active site metal ions and the B2 MBLs only one metal ion (dark grey spheres: zinc ions). The structures of SIE-1, GOB-18, and CSR-1 were each structurally aligned with AIM-1 (shown in white, 20% transparency, light grey zinc ions) for comparison. The figures were generated using the Pymol visualisation software (Schrödinger, 2015). PDB IDs—NDM-1, a B1 MBL: 3S0Z, CphA, a B2 MBL: 1X8G, and the B3 MBLs: AIM-1: 4P62, SIE-1: 7LUU, GOB-18: 5K0W, CSR-1: 6DN4.

**FIGURE 2:**
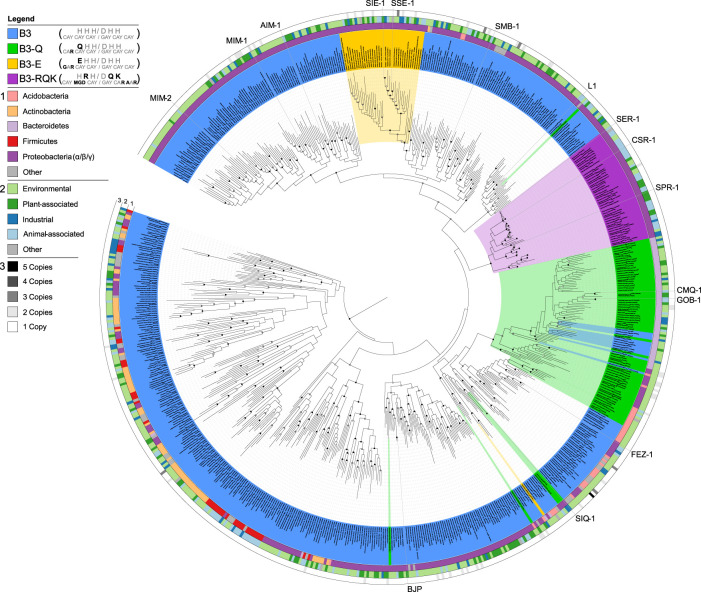
Phylogenetic analysis of MBLs belonging to the B3 subgroup, highlighting the different active site variants. B3 active site variants are highlighted in different colours according to the figure legend in the top left. The inner circle 1) represents the phylum-level affiliations of bacteria containing B3 MBLs; the middle circle 2) represents the source of the B3-containing bacteria; the outer circle 3) represents B3 MBL gene copy number in each genome. Reproduced with permission from Pedroso et al. (2020).

One of the primary mechanisms of resistance to β-lactams, the most widely employed class of antibiotics (Bush and Bradford, 2016), is *via* hydrolysis by β-lactamases, which are divided into four Ambler classes: A, B, C, and D (Bush, 2013). Classes A, C and D are serine-β-lactamases (SBLs), which employ a conserved nucleophilic serine residue during hydrolysis of the β-lactam ring. Class B β-lactamases are metallo-β-lactamases (MBLs) that accommodate one or two zinc ions in their active site, which activate a metal ion-bound hydroxide to initiate β-lactam hydrolysis, though at least some MBLs can employ other divalent metal ions (Bahr et al., 2021). Based on their overall sequences, active site residues, mechanisms, substrate preferences, and phylogenetic relationships, MBLs are further subdivided into three subgroups. While evolutionary analyses indicate that subgroups B1 and B2 form a monophyletic group, B3 MBLs appear to have branched independently, suggesting a convergent evolution to acquire the ability to operate on β-lactam substrates (Hall et al., 2004; Bahr et al., 2021). In addition to being distinct from B1 and B2 MBLs, B3 MBLs also display extensive intra-subgroup diversity, notably with active site variations not observed within the B1 and B2 subgroups (Figures 1, 2) (Hall et al., 2004; Pedroso et al., 2020; Bahr et al., 2021).

The MBL-fold, named after its initial discovery in the MBL BcII (Carfi et al., 1995), has a distinctive αββα core fold (Figure 1) and is observed in a wide diversity of bimetallic metallohydrolases (Bahr et al., 2021). Together, these form the wider MBL superfamily, with members including phosphatases (*e.g.*, nucleases, phytases), pesticide-degrading esterases, sulfatases (Barbeyron et al., 1995; Hagelueken et al., 2006), lactonases (Fernandez et al., 2011; Miraula et al., 2016), dehalogenases (Wang et al., 2010), oxidases (Muok et al., 2019) and the true β-lactam-degrading members, which form only a small subset within the MBL superfamily (Bebrone, 2007; Bahr et al., 2021). Some MBL fold enzymes also display non-hydrolytic reactions, such as the ethylmalonic encephalopathy 1 protein (ETHE1) that catalyses the iron and oxygen-dependent oxidation of glutathione persulfide (GSSH) to give persulfite and glutathione (Tan et al., 2017; Au et al., 2021; Vašková et al., 2023). Interestingly, there is emerging evidence that many of these diverse activities are observed promiscuously throughout the family (Miraula et al., 2016; Diene et al., 2019; Lee et al., 2019; Perez-Garcia et al., 2021). Notably, a number of recent studies have reported promiscuous enzymatic activities in different members of the MBL superfamily, including human nucleases or glyoxalases with β-lactamase activity (Miraula et al., 2016; Tan et al., 2017; Diene et al., 2019; Lee et al., 2019; Au et al., 2021; Perez-Garcia et al., 2021). Promiscuous enzymes with the MBL-fold could thus represent an as-yet overlooked reservoir from which novel sources of antibiotic resistance may arise under appropriate evolutionary pressures.

Most Class B MBLs are extended-spectrum β-lactamases (ESBLs) and effectively inactivate representatives from each of the three most important groups of β-lactam antibiotics, *i.e.*, penicillins, cephalosporins and carbapenems (Hou et al., 2017; Sidjabat et al., 2018; Bahr et al., 2021). Note, however, that monobactams, such as aztreonam, are not MBL substrates. The strong activity of MBLs against carbapenems, or so-called ‘last-resort’ antibiotics, is of particular clinical concern (Hou et al., 2017; Sidjabat et al., 2018; Bahr et al., 2021). Recently, infections from *Pseudomonas aeruginosa*-mediated carbapenem resistance have proliferated and are now a leading cause of death in critically ill and immunocompromised patients (Amsalu et al., 2021; Thaden et al., 2016). The discovery of a highly efficient B3 MBL, Adelaide Imipenemase (AIM-1) in *P. aeruginosa*, with catalytic efficiencies for the inactivation of a broad range of representatives from each of the three major classes of β-lactam antibiotics that is superior to the most effective B1 MBL known to date, NDM-1, demonstrates that the B3 subgroup of MBLs pose a significant but as-of-yet underestimated risk factor to global healthcare (Yong et al., 2012; Selleck et al., 2016; Hou et al., 2017; McGeary et al., 2017). Moreover, while numerous inhibitors for MBLs have been developed (Mohamed et al., 2011; Hussein et al., 2012; McGeary et al., 2014; Arjomandi et al., 2016; McGeary et al., 2017; Krajnc et al., 2019; Palacios et al., 2020; Wachino et al., 2020; Farley et al., 2021) none have yet been used in clinical applications, further exacerbating the threat of MBLs to global health (Mojica et al., 2022). By contrast, potent clinical inhibitors are available for SBLs including the well-established inhibitors clavulanic acid, tazobactam, sulbactam, as well as the more recently developed avibactam and vaborbactam, all of which are used in combination with a β-lactam antibiotic (Hecker et al., 2015; Bush and Bradford, 2016). However, none of the SBL inhibitors are clinically (Bush and Bradford, 2016) effective against MBLs. A major challenge for the development of inhibitors with high specificity for the true MBLs stems from the inherent structural and mechanistic similarity they share with other enzymes from the MBL superfamily [*e.g.*, glyoxalases and some nucleases such as the human enzymes MBLAC1, CPSF73, and HAGH (Pettinati et al., 2016)] as well as enzymes from other families that accommodate bimetallic metal centres that fulfill essential roles in metabolism [e.g. Purple Acid Phosphatases or Phosphodieterases (Mitić et al., 2006; Azevedo et al., 2014)]. The recent discovery of an MBL from the B3 subgroup, CSR-1 from *Cronobacter sakazakii*, that is inhibited by clavulanic acid may thus present a promising avenue to modify existing anti serine-β-lactamase drugs to become broader spectrum therapeutics (Pedroso et al., 2020).

The majority of characterized B3-type MBLs are from environmental bacteria (Hernandez Valladares et al., 2000; Allen et al., 2009; Miraula et al., 2015; Gudeta et al., 2016a; Gudeta et al., 2016b; Pedroso et al., 2017; Rodríguez et al., 2017; Selleck et al., 2020; Wilson et al., 2021) but are increasingly associated with pathogenic organisms as well (*e.g., C. sakazakii*) (Ullah et al., 1998; Yum et al., 2010; Zhou et al., 2019). This mini review highlights recent advances in our understanding of this emerging health threat.

## 2 Discussion

### 2.1 Diversity among antibiotic-degrading MBLs

The B1 subgroup has received by far the most attention amongst studies on the Class B MBLs; it contains some of the most notorious agents that confer resistance to antibiotics. B1 subgroup members include enzymes such as NDM-1, IMP-1, and VIM-2 (Crowder et al., 2006), which have been extensively reviewed previously (Mojica et al., 2016; Bahr et al., 2021). B1-type MBLs are characterised by a conserved HHH/DCH active site motif [i.e., His116, His118, His196 and Asp120, Cys221, His263, as per the BBL numbering scheme (Galleni et al., 2001; Garau et al., 2004)]. The two sets of conserved amino acid side chains provide the ligands that coordinate two zinc ions (*i.e.,* the Zn1 and Zn2 sites, respectively), both of which are required for catalysis (Mitić et al., 2014; Bahr et al., 2021); although it cannot be ruled out that divalent metal ions other than zinc are relevant for MBL activity *in vivo* (Cahill et al., 2016).

The B2-type MBLs constitute the smallest Class B subgroup, of which the best-known member is CphA (Hernandez Valladares et al., 2000). B2 MBLs display the highest degree of substrate selectivity within MBLs, only effectively inactivating carbapenems (Felici et al., 1993; Hernandez Valladares et al., 2000). B2 MBLs are characterised by their NHH/DCH active site motif, corresponding to the Zn1 and Zn2 sites, respectively; however, only one zinc ion, in the Zn2 site, is required for catalysis (Bahr et al., 2021). Indeed, the presence of a Zn(II) in the Zn1 site leads to inhibition (Mitić et al., 2014; Bottoni et al., 2016; Bahr et al., 2021).

The B3 MBL subgroup contains mostly MBLs detected in environmental organisms and is the most divergent group among the Class B β-lactamases, both from phylogenetic and structural perspectives (Pedroso et al., 2020; Fröhlich et al., 2021).

While the B1 and B2 subgroups seem to have a common evolutionary origin, the B3 subgroup likely evolved independently within the overall MBL superfamily, sharing less than 20% sequence similarity with B1 and B2 MBLs (Hall et al., 2004; Bebrone, 2007; Pedroso et al., 2020; Bahr et al., 2021). Furthermore, the diversity internal to the B3 subgroup seems greater than within the B1 and B2 subgroups, with pairwise similarities being as low as 15% (Pedroso et al., 2020). However, B3-type MBLs possess an active site that can accommodate up two Zn(II) ions, and as for B1 MBLs both Zn(II) ions appear to be needed for full catalytic activity (Selleck et al., 2016). The canonical B3 active site motif is HHH/DHH for the Zn1 and Zn2 binding sites, respectively. However, unlike the B1 and B2 subgroups there is considerable diversity within the active site motif of the B3 subgroup (Pedroso et al., 2020). Sequence analyses suggest that there are at least four distinct variants that have evolved within the B3 clade, *i.e.*, the canonical form with the HHH/DHH active site motif, and three less abundant forms (Figure 2) (Pedroso et al., 2020). Two of these variants have a substitution of residue 116 of the Zn1 site, replacing the canonical histidine by either a glutamine (Q) or a glutamic acid (E), resulting in the B3-Q (**Q**HH/DHH) or B3-E (**E**HH/DHH) active site motifs, respectively (Figures 1, 2). The glutamine substitution, in contrast to the glutamate mutation, appears to have occurred independently on multiple occasions within the B3 subfamily (Figure 2) (Pedroso et al., 2020). The least abundant and most intriguing variant, characterised by the B3-RQK active site motif, has three mutations relative to the canonical HHH/DHH motif, *i.e.*, a histidine to arginine of residue 118 of the Zn1 site, and histidine to glutamine and histidine to lysine in residues 121 and 263 of the Zn2 site, respectively, resulting in the H**R**H/D**QK** active site motif (Vella et al., 2013; Pedroso et al., 2020) (Figures 1, 2). This variant appears to form a monophyletic group, having evolved from a single evolutionary event within the B3 MBL subgroup (Figure 2) (Pedroso et al., 2020).

### 2.2 Structures and catalytic properties of MBLs from the B3 subgroup

a) B3 variants with the canonical HHH/DHH motif. The most studied representatives from the B3 MBL subgroup are L1 from *S. maltophilia* (Simm et al., 2002; Aitha et al., 2016; Kim et al., 2020) and AIM-1 from *P. aeruginosa* (Selleck et al., 2016), with the latter posing a major threat to healthcare due to its high catalytic efficiency towards a broad range of β-lactam antibiotics (Supplementary Table S1). The metal ion in the Zn1 site is tetrahedrally coordinated by the three histidine ligands of the active site motif, as well as the oxygen from the hydroxide that in the resting enzyme bridges the two metal ions and which acts as the nucleophile that initiates the hydrolysis of the β-lactam bond. The Zn2 site usually adopts a distorted trigonal bipyramidal geometry where His121 and His263 from the active site motif and the metal ion-bridging nucleophilic hydroxide form the equatorial plane, and Asp120 and an additional water ligand occupy axial positions (Figure 1). Overall, the active site geometry of AIM-1 is similar to that of most of the characterised B1-type MBLs, including that of NDM-1 (King and Strynadka, 2011) or IMP-1 (Concha et al., 2000; Yamaguchi et al., 2021).

MBLs from the B3 subgroup have received far less attention than representatives from the B1 subgroup, largely because they have historically been less often associated with human pathogens. However, they are increasingly being identified in clinical settings and likely pose an underestimated threat to global health (Ullah et al., 1998; Yum et al., 2010; Zhou et al., 2019). For example, AIM-1, originally isolated from a patient from a hospital in Adelaide, Australia, is expressed by bacteria such as *P. aeruginosa* and *K. pneumoniae* (Zhou et al., 2019) and is located on a mobile genetic element (Yong et al., 2012). A recent shotgun genomic sequencing and phylogenetics analysis of wastewater samples from different locations has revealed that, although the gene encoding for AIM-1 may have originated from the non-pathogenic, environmental species *Pseudoxanthomonas mexicana*, it is now widely distributed across the globe (Amsalu et al., 2021).

Furthermore, AIM-1 is highly efficient in inactivating representatives from each of the three major groups of β-lactam antibiotics. In fact, it is considerably more reactive against most of those substrates than NDM-1, one of the most effective agents in antibiotic-resistant isolates around the globe (World Health Organization, 2014; Mojica et al., 2022). In particular, AIM-1 is highly efficient in inactivating “last-resort” carbapenems, with *k*_cat_/*K*_M_ ratios as high as 18 s^-1^µM^-1^, nearly 100-fold higher than the corresponding values reported for NDM-1 (Supplementary Table S1). Mechanistic studies have shown, that like the B1 MBLs, AIM-1 operates most effectively with two Zn(II) bound to the active site (Selleck et al., 2016).

b) B3 variants with the QHH/DHH motif (B3-Q). The best characterised members of the B3-Q MBL variants are the GOB family of MBLs, although several other B3 members have been recovered from environmental soil metagenomes (*e.g.*, LRA-12 or PEDO-1) (Gudeta et al., 2016a; Gudeta et al., 2016b; Rodríguez et al., 2017). GOB-1 was discovered in the opportunistically pathogenic bacterial species *E. meningoseptica* (Horsfall et al., 2011), but numerous GOB variants have since been discovered in *E. meningoseptica* and other *Elizabethkingia* species (Yum et al., 2010; Pedroso et al., 2020; Bahr et al., 2021).

Structural data are available only for one GOB variant, GOB-18 (PDB: 5K0W), and provide evidence that the histidine to glutamine substitution in position116 of the Zn1 motif does not greatly affect the geometry of the active site when compared with that of AIM-1 (Morán-Barrio et al., 2016). However, a noteworthy difference between GOB-18 and AIM-1 is the inversion in the coordination geometries of the Zn1 and Zn2 sites within GOB-18, with Zn2 adopting a tetrahedral and the Zn1 a trigonal bipyramidal geometry (including an axial water ligand) (Figure 1) (Morán-Barrio et al., 2016; Wilson et al., 2021). Similarly to AIM-1, GOB-1 is also effective against a broad range of β-lactam antibiotics, in particular carbapenems, with *k*_cat_/*K*_M_ ratios as high as 8 s^-1^µM^-1^ (Supplementary Table S1). It is reported that GOB-18 may occur both in mono- and bi-metallic form, similar to the B1-type MBL BsII from *Bacillus subtilis*, and it may thus be possible that GOB MBLs are active in both states (Morán-Barrio et al., 2007; Lisa et al., 2010). Indeed, the monometallic form may be particularly active against carbapenem substrates, using a mechanism similar to B2-type MBLs that almost exclusively operate on these substrates (Garau et al., 2005; Fonseca et al., 2011).

c) 3 variants with the EHH/DHH motif (B3-E). Only one member of the B3-E variants has been characterised to date, SIE-1 from *Sphingobium indicum* (Wilson et al., 2021), but sequences of other putative B3 MBLs sharing this active site motif have been reported (Pedroso et al., 2020; Bahr et al., 2021). The replacement of the histidine in position 116 of the canonical Zn1 motif by a glutamate does not lead to significant structural changes when compared to AIM-1, preserving a tetrahedral Zn1 and a trigonal bipyramidal Zn2 site (Figure 1) (Wilson et al., 2021). The largest structural difference between the active sites of SIE-1 and canonical B3 MBLs (*e.g.*, AIM-1) or B3-Q (*i.e.*, GOB-18) is that the Zn1-Zn2 distance is longer in SIE-1 (3.75Å) than in AIM-1 (3.48Å) or GOB-18 (3.5Å) (Wilson et al., 2021).

Like other B3 MBLs, SIE-1 is catalytically active against all major classes of β-lactams (Supplementary Table S1). While generally less efficient than the canonical AIM-1 or the B3-Q enzyme GOB-18, SIE-1 has catalytic parameters comparable to those of the B1-type MBL NDM-1 (Wilson et al., 2021). Interestingly, SIE-1 shows increased selectivity toward cephalosporin substrates (with *k*_cat_/*K*_M_ ratios as high as ∼1.3 s^-1^µM^-1^) and is thus more efficient against such substrates than penicillins, an infrequent characteristic amongst B3 MBLs (Wilson et al., 2021) (Supplementary Table S1). From the limited data currently available, it appears that B3-E variants likely employ a mechanism involving two Zn(II) ions in the active site, similar to AIM-1 (Wilson et al., 2021).

d) B3 variants with the HRH/DQK motif (B3-RQK). The B3-RQK subfamily is the most divergent and least represented among the B3 MBL variants, with substitutions in both the Zn1 and Zn2 sites (Figure 2). To date representatives of the B3-RQK variants have been discovered in and characterised from *Serratia proteamaculans* (SPR-1) (Vella et al., 2013), *C. sakazakii* (CSR-1) (Pedroso et al., 2020) and *Salmonella enterica* (SER-1) (Pedroso et al., 2020). Members of the B3-RQK class are not only unusual because they have the largest number of substitutions in their active sites, but also because two of the canonical histidine residues were replaced by arginine and lysine residues, both very rare ligands in metal ion-dependent enzymes. Due to their high pK_a_ values, these residues are likely to be protonated at pH values optimal for MBL activity, and thus unlikely to coordinate to metal ions effectively. This interpretation is in agreement with initial studies of SPR-1, which indicated that it may have only one Zn(II) bound in the resting state, but that a catalytically active di-Zn(II) centre is formed upon the addition of a substrate (Vella et al., 2013). This substrate-promoted activation mechanism is similar to that proposed for the pesticide-degrading diesterase GpdQ (Hadler et al., 2009; Hadler et al., 2010; Paul et al., 2018; Sharma et al., 2019; Sharma et al., 2020) and indicates an inherent plasticity in the mechanism and potentially the functionality of these enzymes.

Indeed, a high-resolution structure and binding studies of the B3-RQK enzyme CSR-1 reveals that its active site has a strongly decreased metal ion affinity when compared to AIM-1 (Pedroso et al., 2020). Furthermore, while CSR-1 is active against substrates from each of the three major groups of β-lactam antibiotics, it is considerably less efficient than other MBLs (Supplementary Table S1). It has been demonstrated that, at least for some sets of MBLs, that the active site metal ion affinity correlates with catalytic efficiency, in particular for the Zn2 site. In the case of CSR-1 replacement of its arginine in position 118 by the canonical histidine enhances metal ion binding affinity at the Zn1 site with a modest improvement in catalytic efficiency (Supplementary Table S2). However, replacing both the glutamine and lysine residues in the CSR-1 Zn2 site with their canonical histidine counterparts enhances the affinities of both Zn(II) ions and drastically improves the catalytic efficiency to a level comparable to that of other B3 MBLs (Supplementary Table S2). Given that B3-RQK variants evolved from within the B3 MBL subgroup (Figure 2), it is possible that these enzymes indeed represent a functional adaptation, even though no alternative substrates for them have yet been identified.

### 2.3 Inhibition of B3-RQK MBLs may provide an avenue to combat antibiotic resistance

Given the rise of antibiotic resistance, the lack of clinical inhibitors for MBLs is of pressing concern. A range of SBL inhibitors have been tested to no avail against numerous MBLs, such as penams (*e.g.*, sulbactam and tazobactam), diazabicyclooctanones (*e.g.*, avibactam and relebactam) (Wang et al., 2016; Lang Pauline et al., 2021), and some boronates (*e.g.*, vaborbactam) (Bush and Bradford, 2016; Cahill et al., 2017; Bahr et al., 2021). Some newer agents such as next-generation boronates (*e.g.*, taniborbactam or xeruborbactam, which are being tested in clinical trials) (Krajnc et al., 2019; Hamrick et al., 2020; Hecker et al., 2020; Liu et al., 2020; Bahr et al., 2021; Brem et al., 2022; Yang et al., 2023), thiol-based compounds (Brem et al., 2016; Tehrani and Martin, 2017) [*e.g.*, captopril (Heinz et al., 2003)], carboxylate-based compounds (Concha et al., 2000; Hinchliffe et al., 2018; Brem et al., 2022), phosphonate-based compounds (Yang et al., 2013; Pemberton et al., 2019), or pyrazoles (Alam, 2022) hold some promise as MBL inhibitors. The recent observation that the commonly used serine-β-lactamase inhibitor clavulanic acid (a clavam) inhibits B3-RQK MBLs may be leveraged to broaden the application scope of this widely used therapeutic. Clavulanic acid is a known inhibitor of serine-β-lactamases (Drawz and Bonomo, 2010), especially those belonging to Classes A and D with K_i_ values ranging from 20 to 200 µM (Pedroso et al., 2020).

While ineffective against all known MBLs associated with antibiotic resistance in clinical isolates, clavulanic acid inhibits B3-RQK with a K_i_ of 200–350 µM (Pedroso et al., 2020). A combination of docking and mutagenesis studies with CSR-1 demonstrated that the lysine residue in position 263 of the Zn2 active site motif may play a crucial role in the binding of clavulanic acid by enabling it to outcompete the catalytically essential Zn(II) in this site (Pedroso et al., 2020). Replacing this lysine with the canonical histidine favours the binding of the metal ion and thus prevents inhibition by clavulanic acid. Considering the overall similarity of the active site geometry in the metal binding pocket of MBLs it may thus be possible to modify clavulanic acid so that its affinity to the Zn2 site will be greater than that of the metal ion.

## 3 Future perspectives

MBLs have emerged as a major threat to global health, compounded by a lack of clinically suitable inhibitors. The most prevalent MBL subgroup associated with antibiotic resistance is B1, which have highly conserved active site geometries around the catalytically essential Zn(II) ions. However, in recent years an increasing number of MBLs from the B3 subgroup have been identified and characterised. To date, most B3 MBLs are associated with environmental microorganisms that are not pathogenic for humans, but a growing number have been found in more concerning bacteria such as *K. pneumoniae*.

Recent studies have shown that B3 MBLs are evolutionarily related to enzymes with distinct functions, in particular different types of nucleases (Diene et al., 2019), and they have also been identified in several viral genomes. It is thus plausible that B3 MBLs have evolved from a different ancestral functionality and acquired β-lactamase activity in response to environmental pressure. Considering the abundance of such B3 MBLs in diverse microbial organisms it may be only a matter of time before more of these agents accumulate in human pathogens, thus presenting a new threat to healthcare. It is therefore imperative to think about inhibition strategies to combat them now.

Furthermore, the B3 subgroup is more diverse in terms of active site geometry, mechanism, reactivity, substrate selectivity, and inhibition than the B1 and B2 MBL subgroups. There is a thus a risk that inhibitors developed solely against the currently most important B1 MBL subgroup, which contains the notorious but relatively recently discovered NDM (discovered, 2009), VIM (1999), and IMP (1991) MBLs” to “recently discovered NDM- (discovered, 2009), VIM- (1999), and IMP-type (1991) MBLs, will be become less effective due to a future rise in B3 (and B2) MBLs, against which they may not be active. Interestingly, one active site variant among the B3 MBLs is inhibited by the widely used SBL inhibitor clavulanic acid, by a mechanism involving displacement of the Zn2 metal ion in the active site for which interactions with residue 263 appear to be particularly important. This observation suggests that it may be possible to modify clavulanic acid or related compounds to possess enhanced affinity for the Zn2 site. Along with reports on bicyclic boronates (Cahill et al., 2017; Krajnc et al., 2019), it may be within reach to generate very broad spectrum β-lactamase inhibitors.
