# Supplementary material for: Structure, function, and evolution of metallo-β-lactamases from the B3 subgroup—emerging targets to combat antibiotic resistance
Source: Front Chem. 2023 Jun 20;11:1196073. doi: 10.3389/fchem.2023.1196073 (PMC10318434; doi:10.3389/fchem.2023.1196073)
Supplement: Supplementary file 1 [file Table1.pdf]

Supplementary Material

Structure, Function and Evolution of Metallo- β-Lactamases from the B3 Subgroup – Emerging Targets to Combat Antibiotic Resistance

Stefan Krco[1,2 †], Samuel Davis[2 †], Pallav Joshi[1], Liam Wilson[3], Marcelo Monteiro Pedroso[1,2], Andrew Douw[1], Christopher Schofield[3], Philip Hugenholtz[1,2], Gerhard Schenk[1,2,4,5]*, & Marc T. Morris[1,2]*

*** Correspondence:**

Marc T. Morris: marc.morris@uq.edu.au

Gerhard Schenk : schenk@uq.edu.au

1 Supplementary Tables

See next page

Table S1: Kinetic parameters of representative MBLs for reactions with select β-lactam substrates from the three major groups used in clinical settings. k_{cat} values are in s^{-1}, K_M in μM and k_{cat}/K_M are in $s^{-1}\mu M^{-1}$. [a]Yong et al., (2009), [b]Marcoccia et al., (2016), [c]Yong et al., (2012) & Selleck et al., (2016), [d]Horsfall et al., (2011), [e]Wilson et al., (2021), [f]Pedroso et al., (2020).

		**NDM-1**[1]			**AIM-1**[c, 2]			**GOB-1**[d, 3]			**SIE-1**[e, 4]			**CSR-1**[f, 5]		
		B1: HHH/DCH			B3: HHH/DHH			B3-Q: QHH/DHH			B3-E: EHH/DHH			B3-RQK: HRH/DQK		
**Substrate**		k_{cat}	K_M	k_{cat}/K_M	k_{cat}	K_M	k_{cat}/K_M	k_{cat}	K_M	k_{cat}/K_M	k_{cat}	K_M	k_{cat}/K_M	k_{cat}	k_{cat}	k_{cat}/K_M
Penicillins	Penicillin G	11	16[a]	0.68	778	31	25	630 ± 10	190 ± 10	3.4	360 ± 20	450 ± 50	0.79	17 ± 3	250 ± 10	0.07
	Ampicillin	15	22[a]	0.66	594	41	14	-	-	-	100 ± 20	1900 ± 600	0.05	11 ± 2	178 ± 31	0.06
Carbapenems	Meropenem	12	49[a]	0.25	1000	163	6.1	170 ± 3	22 ± 1	8.0	92 ± 6	100 ± 20	0.92	6.0 ± 3	310 ± 11	0.02
	Imipenem	20	95[a]	0.21	1700	97	18	85 ± 2	13 ± 1	6.5	27 ± 3	250 ± 60	0.11	8.0 ± 1	276 ± 17	0.03
Cephalosporins	Cephalothin	4	10[a]	0.4	529	38	14	32 ± 0.4	7.9 ± 0.5	4.0	16 ± 1	13 ± 1	1.26	-	-	-
	Cefuroxime	5	8[a]	0.61	292	29	10	-	-	-	25 ± 2	25 ± 5	0.99	1.0 ± 0.9	110 ± 8.0	0.01
	Nitrocefin	10	15[b]	0.67	240	96	2.5	14 ± 3	16	0.87	29 ± 3	32 ± 5	0.90	2 ± 0.3	74	0.03

1 *Klebsiella pneumoniae*
2 *Pseudomonas aeruginosa*
3 *Elizabethkingia meningoseptica*
4 *Sphingobium indicum*
5 *Cronobacter sakazakii*

Table S2: Kinetic parameters of CSR-1, a B3-RQK MBL, and three CSR-1 mutants for reactions with select β-lactam substrates from the three major groups used in clinical settings. k_{cat} values are in s^{-1}, K_M in µM and k_{cat}/K_M are in $s^{-1}µM^{-1}$. Data was obtained from Pedroso et al., (2020).

Substrate		**CSR-1** HRH/DQK			**CSR-1$_{R118H}$** HHH/DQK			**CSR-1$_{Q121H, K263H}$** HRH/DHH			**CSR-1$_{R118H, Q121H, K263H}$** HHH/DHH		
		**k_{cat}**	**K_M**	**k_{cat}/K_M**	**k_{cat}**	**K_M**	**k_{cat}/K_M**	**k_{cat}**	**K_M**	**k_{cat}/K_M**	**k_{cat}**	**K_M**	**k_{cat}/K_M**
Penicillins	Penicillin G	17 ± 3	250 ± 10	0.07	12 ± 7	380 ± 27	0.03	100 ± 9	154 ± 8	0.65	78 ± 2	75 ± 5	1.04
	Ampicillin	11 ± 2	178 ± 31	0.06	23 ± 8	200 ± 21	0.12	89 ± 7	110 ± 21	0.81	170 ± 9	98 ± 11	1.74
Carbapenems	Meropenem	6.0 ± 3	310 ± 11	0.02	12 ± 2	132 ± 7	0.09	152 ± 9	144 ± 31	1.1	251 ± 3	178 ± 12	1.41
	Imipenem	8.0 ± 1	276 ± 17	0.03	10 ± 2	87 ± 8	0.11	27 ± 7	130 ± 45	0.21	183 ± 10	211 ± 11	0.87
Cephalosporins	Cefuroxime	1.0 ± 0.9	110 ± 8.0	0.01	1.7 ± 0.9	272 ± 33	0.01	60 ± 3	181 ± 13	0.33	110 ± 7	380 ± 33	0.29
	Cefoxitin	0.7 ± 0.6	189 ± 15	<0.01	2 ± 0.7	95 ± 21	0.02	5 ± 1	167 ± 21	0.03	21 ± 8	279 ± 21	0.08

References

Horsfall, L.E., Izougarhane, Y., Lassaux, P., Selevsek, N., Liénard, B.M.R., Poirel, L., et al. (2011). Broad antibiotic resistance profile of the subclass B3 metallo-β-lactamase GOB-1, a di-zinc enzyme. *The FEBS Journal* 278(8)**,** 1252-1263. doi: https://doi.org/10.1111/j.1742-4658.2011.08046.x.

Marcoccia, F., Bottoni, C., Sabatini, A., Colapietro, M., Mercuri Paola, S., Galleni, M., et al. (2016). Kinetic Study of Laboratory Mutants of NDM-1 Metallo-β-Lactamase and the Importance of an Isoleucine at Position 35. *Antimicrobial Agents and Chemotherapy* 60(4)**,** 2366-2372. doi: 10.1128/AAC.00531-15.

Pedroso, M.M., Waite, D.W., Melse, O., Wilson, L., Mitić, N., McGeary, R.P., et al. (2020). Broad spectrum antibiotic-degrading metallo-β-lactamases are phylogenetically diverse. *Protein & cell* 11(8)**,** 613-617. doi: 10.1007/s13238-020-00736-4.

Selleck, C., Larrabee, J.A., Harmer, J., Guddat, L.W., Mitić, N., Helweh, W., et al. (2016). AIM-1: An Antibiotic-Degrading Metallohydrolase That Displays Mechanistic Flexibility. *Chemistry – A European Journal* 22(49)**,** 17704-17714. doi: https://doi.org/10.1002/chem.201602762.

Wilson, L.A., Knaven, E.G., Morris, M.T., Monteiro Pedroso, M., Schofield, C.J., Brück, T.B., et al. (2021). Kinetic and Structural Characterization of the First B3 Metallo-β-Lactamase with an Active-Site Glutamic Acid. *Antimicrobial Agents and Chemotherapy* 65(10)**,** e00936-00921. doi: 10.1128/AAC.00936-21.

Yong, D., Toleman, M.A., Giske, C.G., Cho, H.S., Sundman, K., Lee, K., et al. (2009). Characterization of a new metallo-beta-lactamase gene, bla(NDM-1), and a novel erythromycin esterase gene carried on a unique genetic structure in Klebsiella pneumoniae sequence type 14 from India. *Antimicrob Agents Chemother* 53(12)**,** 5046-5054. doi: 10.1128/aac.00774-09.

Yong, D., Toleman Mark, A., Bell, J., Ritchie, B., Pratt, R., Ryley, H., et al. (2012). Genetic and Biochemical Characterization of an Acquired Subgroup B3 Metallo-β-Lactamase Gene, blaAIM-1, and Its Unique Genetic Context in Pseudomonas aeruginosa from Australia. *Antimicrobial Agents and Chemotherapy* 56(12)**,** 6154-6159. doi: 10.1128/AAC.05654-11.
